# Doctor

**Published:** 1871-01

**Authors:** 


### DOCTOR.

In Webster's Dictionary, we read that the title "doctor" is only given to one who is an instructor, or one who has received the highest degree of a faculty—one who has received a diploma from a university or college, authorizing him to teach and practice; as a *doctor* of divinity, of law, of medicine, of music, of philosophy, &c. But it would seem that there are many kinds of doctors now-a-days, that old Noah Webster never dreamed of. If a person engages in the practice of dentistry, having qualified himself by working six months in some dental office, he seeks to be styled "doctor," and forthwith attaches this title to his name and card.

A young man who has officiated as clerk behind some druggist's counter, for the space of three months, also becomes a "doctor," and even does not blush when so called. The druggist himself is a "doctor," no matter whether he is a graduate of some pharmaceutical college or not,—he's a "doctor," for so people insist upon calling him.

This habit of styling druggists and their clerks "doctor," has led to many fatal errors. People are led to believe that they really *are* doctors, and often seek advice and medicines at their hands. We know of several druggists, not physicians, who are constantly in the habit of prescribing over their counters to patients who suppose them to be *bona fide* physicians. We have known of one or two almost fatal mistakes from this abominal practice, and it is high time that something should be done to suppress it. If physicians would refuse to patronize, or allow their prescriptions to be prepared by such druggists, one step to'rd removing the evil, would be well taken. It is not only an injustice to the physician, but a fraud perpetrated upon the patient.

